# Gelling Characteristics of Emulsions Prepared with Modified Whey Protein by Multiple-Frequency Divergent Ultrasound at Different Ultrasonic Power and Frequency Mode

**DOI:** 10.3390/polym14102054

**Published:** 2022-05-18

**Authors:** Yu Cheng, Georgina Benewaa Yeboah, Xinyi Guo, Prince Ofori Donkor, Juan Wu

**Affiliations:** 1 School of Food and Biological Engineering, Jiangsu University, 301 Xuefu Road, Zhenjiang 212013, China; ama.benewaa@yahoo.com (G.B.Y.); 3190910029@stmail.ujs.edu.cn (X.G.); 2 Institute of Food Physical Processing, Jiangsu University, 301 Xuefu Road, Zhenjiang 212013, China; 3 Food Nutrition Science Centre, School of Food Science and Biotechnology, Zhejiang Gongshang University, Hangzhou 310012, China; princeoforidonkor@yahoo.com

**Keywords:** dual-frequency, hardness, storage modulus, microstructure, water holding capacity, digestion

## Abstract

The effect of ultrasonic frequency mode (mono, dual and tri-frequency) and ultrasonic power (0–300 W) on structural properties (intrinsic fluorescence and sulfhydryl content) of whey protein was studied. Emulsions prepared with modified whey protein were used to form the heat-set gels, and the properties of whey protein emulsion gels (WPEG) and their digestion were investigated. The textural and rheological properties of WPEG prepared using whey protein pretreated by mono and dual-frequency ultrasound at the power between 180–240 W were enhanced, while those of WPEG prepared with whey protein pretreated by triple-frequency above the power of 180 W were declined. WPEG prepared using whey protein pretreated by dual-frequency ultrasound (DFU) with the power of 240 W had the highest hardness and storage modulus which were 3.07 and 1.41 times higher than the control. The microstructure of WPEG prepared using DFU pretreated whey protein showed homogeneous and denser networks than those of the control according to the results of confocal laser scanning microscope (CLSM). The modification in the microstructure and properties of the WPEG prepared using DFU pretreated whey protein delayed the protein disintegration during the first 30 min of gastric digestion when compared with control. Whereas the release rate of free amino group of the WPEG prepared using whey protein modified by ultrasonic pretreatment increased during the intestinal phase when compared with that of control. The results indicated that using dual-frequency ultrasound to modify whey protein is more efficient in improving the properties of WPEG, and ultrasonic power should be considered during the application of ultrasound pretreatment in producing protein gels. The fine network of WPEG prepared with whey protein pretreated by ultrasound resulted in better hardness and storage modulus. Partially unfolding of the protein induced by ultrasound pretreatment might make the whey protein more susceptible to the digestive enzyme. Our results could provide new insights for using ultrasound as the potential processing tool on designing specific protein emulsion gels as the delivery system for nutrients.

## 1. Introduction

Food products with varying structures are being formulated to control digestion and nutrient release, as the structure of food has been shown to play an important role in digestion [1]. Emulsion gels are soft-solid structures that exhibit emulsion and gel properties simultaneously. These food systems are employed to manufacture foods such as yoghurts, soft cheese, mayonnaise, and dairy desserts, and gained increasing attentions by serving as encapsulation media for transporting bioactive compounds and nutraceuticals [2,3]. The structure of emulsion gels serves as a medium by which they protect bioactive substances against the harsh conditions in gastrointestinal tract. The property and the structure of emulsion gels are critical for nutrient delivery, because the structure of the gel network affects enzyme accessibility during digestion [4,5,6]. Previous studies indicated that emulsion gels with desired structure can be developed to control nutrient release by manipulating protein structure [7], and detailed investigations on the effect of structural and rheological properties on digestion of whey protein emulsion gels (WPEG) have been conducted [8,9].

Ultrasound is a green technology that has been used in the food industry for its ability to modify the structure, physicochemical properties and functionality of proteins [10]. These structural changes result from the generation and collapse of microbubbles in the protein due to pressure fluctuations produced by acoustic cavitation [11]. The ultrasound improved properties of protein, such as particle size, emulsion stability, hydrophobicity, viscosity, gelation and solubility [12,13]. In gels, the ultrasound might improve their water holding capacity (WHC), storage modulus, hardness and microstructure [12,14,15].

Although a significant number of studies focused on mono-frequency ultrasound (MFU), it has been proven that multi-frequency ultrasound could amplify the effect of ultrasound [16,17]. Dual and triple frequency ultrasound have two and three ultrasonic waves with different frequency respectively. Superposition of waves can generate homogeneous ultrasound field with higher cavitational effect and waveform with wider range to avoid the formation of standing waves [18] when compared with mono frequency ultrasound. It can intensify the mass transfer and result in more sensitive protein structure to the enzymes. As a result, dual-frequency ultrasound (DFU) and triple frequency ultrasound (TFU) yielded better results than MFU during extraction [10] and protein hydrolysis [19]. Cheng et al. [15] compared the effect of MFU and DFU modifying whey protein on mechanical properties of WPEG and confirmed that DFU treatment was associated with better WHC, hardness and rheological properties. At present, mono-frequency probe ultrasound (MFPU) was the energy-gathered ultrasound that used in most of the researches on preparing protein gels. However, probe ultrasound has its drawbacks that the samples might be contaminated by the metal probe, and the region which ultrasonic energy can reach is limited. The divergent ultrasound, which will not contact direct with the samples, was selected in this study for better industrial application in processing protein solutions.

Besides the ultrasonic frequency mode, ultrasonic power is an important factor influencing the degree of modification in proteins [20,21,22]. It was discovered that increasing ultrasonic power (0–600 W) improved the structure and emulsifying properties of whey protein [23]. In milk gels, intermediate power reduced gelation time, although storage modulus was reported to be increased with power [24]. However, there is limited report on the effect of ultrasonic power on the gel properties of WPEG, and the effect of multiple-frequency ultrasound treatment on simulated digestion of WPEG is less studied.

Therefore, this study aimed to investigate multi-frequency divergent ultrasound mode (mono-frequency ultrasound at 20 kHz, dual -frequency ultrasound at 20/35 kHz and tri-frequency at 20/35/50 kHz) and ultrasonic power (0–300 W) as key processing factors on modifying the properties of WPEG. Moreover, the impacts of the ultrasound modification on WPEG during in vitro gastric digestion were investigated in order to design specific protein emulsion gels as the delivery system for nutrients.

## 2. Materials and Methods

### 2.1. Materials

Whey protein isolate (WPI) (92 wt% protein, 1.5 wt% lipid, 1.5 wt% carbohydrate and sodium 0.895 wt%) was purchased from Hilmar Company (Hilmar, CA, USA). Nile red, fast green, pepsin and pancreatin were purchased from Sigma-Aldrich (St. Louis, MO, USA). Soybean oil was acquired from a supermarket in Zhenjiang, China, and used without further processing. All other chemicals/reagents were of analytical grade.

### 2.2. Whey Protein Solution Preparation and Ultrasound Pretreatment

WPI solution (10%, *w*/*v*) was prepared with phosphate buffer (pH 7.0, NaCl 50 mM) and stirred (400 rpm) for 4 h at room temperature, following by ultrasonic modification. Ultrasound pretreatment on WPI solution was carried out with a triple frequency (20, 35, 50 kHz) ultrasonic bath (300 W) developed by our team and manufactured by Meibo Biotechnology Co. Ltd. (Zhenjiang, China). Our team’s preliminary experiments gave the ultrasonic time for the three frequencies as follows: 20 kHz for 30 min, 20/35 kHz for 15 min and 20/35/50 kHz for 10 min at 25 ± 2 °C with a pulse on and off time of 5 s and 2 s, respectively. All experiments with the dual and triple frequency were carried out in simultaneous mode. Pretreatment was done at five different power levels (60, 120, 180, 240, 300 W), representing power densities of 20, 40, 60, 80 & 100% of the equipment’s full power capacity, for all three frequencies at their optimum sonication time.

### 2.3. Intrinsic Fluorescence Spectrum

Intrinsic fluorescence of samples was determined using Cary Eclipse fluorescence spectrophotometer (Varian Inc., Palo Alto, CA, USA) equipped with a 1 cm path length cell. Intrinsic fluorescence of sonicated and non-sonicated WPI solution (1 mg/mL) was determined using a 5 nm slit excitation at an excitation wavelength of 280 nm and an emission wavelength range of 290–400 nm. Tests were carried out in triplicates.

### 2.4. Sulfhydryl (SH) Content

The Ellman (DTNB) reagent method [25] was used with some modifications to determine SH. The protein samples were dissolved in buffer 1 (standard Tris-glycine containing 0.086 mol/L Tris, 0.09 mol/L Gly, 4 mmol/L EDTA, pH 8) and buffer 2 (8 mM Urea dissolved in buffer 1) to a concentration of 5 mg/mL. Buffers 1 and 2 were used to determine total and free SH respectively. DTNB (0.02 mL) was then added to 2 mL of protein-buffer mixture. The mixture was stored in the dark in a water bath at 25 °C for 30 min. Absorbance was then measured at 412 nm with buffer and DTNB as blank. Sulfhydryl content was calculated as: SH (μmol/g) = (73.53 × A_412_ × D)/C, where A_412_ is the absorbance value; D is the dilution factor; C is the protein concentration.

### 2.5. Emulsion Preparation

Emulsions were prepared according to Cheng et al. [15] with some modifications. Using a ratio of 4:1 (protein solution: oil), the ultrasound pretreated whey protein solution was mixed with soybean oil (20 wt%) by homogenization using a high-speed blender (HG-15A, DAIHAN Scientific Co. Ltd. Daejeon, Korea) at 10,000 rpm for 2 min. The pre-emulsions were passed through a two-stage high-pressure homogenizer (AH-BASIC, ATS Engineering Inc., Brampton, ON, Canada) at the first pressure of 300 bar and the second stage pressure of 50 bar with two passes to form emulsions. Emulsions were stored at 4 °C until use.

### 2.6. Droplet Size and Zeta Potential Measurement

Freshly prepared emulsions diluted to an oil concentration of 0.05% were used for zeta potential and droplet size measurements in a particle size analyser (Anton Paar Litesizer 500 light scattering instrument, Anton Paar GmbH, Graz, Austria).

### 2.7. Rheological Properties

A DHR-1 rheometer (TA Instruments, New Castle, DE, UK) built with a temperature control system was used to study rheological properties through heating and cooling of the prepared emulsions. Measurements were taken using a stainless steel oscillating bob and stable cup in a concentric cylinder. The cup was filled with 20 mL of emulsions and covered with 1 mL of silicone oil to stop evaporation. Gelation was heat-induced in an oscillation mode using 0.5% strain and frequency of 1 Hz with four steps; heating samples from 25–90 °C at 5 °C/min; holding at 90 °C for 1800 s; cooling from 90–25 °C and holding at 25 °C for 900 s. For viscosity, approximately 1.2 mL of whey protein emulsions was used on a 40 mm plate in a flow-ramp mode.

### 2.8. Emulsion Gel Formation

Amount of 5 mL and 15 mL prepared emulsions were poured into 16.5 cm (internal diameter) × 50 cm high and 20 mm diameter × 30 mm high glass tubes respectively. The tubes were covered and heated at 90 °C for 30 min in a water bath. Emulsion gels were kept at 4 °C for 24 h before analysis.

### 2.9. Texture Profile Analysis (TPA)

Emulsion gels (from 20 mm diameter × 30 mm high glass tubes) were carefully removed without breaking and subjected to Texture profile analysis (TPA) with a Texture Analyser (TA-XT Plus, Stable Microsystems, Surrey, UK). With an aluminium cylindrical probe of 50 mm diameter, compression strain of 30% and crosshead speed of 1 mm/s, at least 6 replicates were taken for each treatment.

### 2.10. Water Holding Capacity (WHC)

The WHC was carried out after Cheng et al. [15] with some modifications. Whey protein emulsion gels (from 16.6 cm × 50 cm glass tubes) were weighed, wrapped with filter paper, and placed on cotton wool stuck in 50 mL centrifuge tubes. They were centrifuged (TGL-16G centrifuge, Anting Scientific Instrument Co. Ltd., Shanghai, China) at 2000× *g* for 20 min at 20 °C. The filter paper was then removed, and gels were reweighed. WHC of gels were expressed as a percentage of the difference between the weights.

WHC (%) = 100 × (W_1_ − W_2_)/W_1_

where W_1_ is the weight before centrifugation while, W_2_ is the weight after centrifugation.

### 2.11. Confocal Laser Scanning Microscope (CSLM)

Gel samples (1 mm thick) were stained with 10 µL Fast green and 20 µL Nile red for overnight. The stained gels were placed on a concave slide and covered with slip. Images were obtained at a 40× magnification using Leica TCS SP5. The images were analyzed using ImageJ 1.50i (National Institute of Health, Bethesda, MD, USA).

### 2.12. Simulated Digestion

Simulated digestion was performed after Minekus et al. [26] with modifications. Experiment was repeated at least twice. WPEG was blended to simulate oral chewing for a minute and then mixed with SSF (pH 7) in a 1:1 ratio without amylase. This mixture was kept at 37 °C for 2 min with gentle shaking. The gastric digesta was mixed with SGF in a 1:1 ratio and pH was adjusted to 3.0 with 1 M hydrochloric acid. CaCl_2_ (0.3 M) was stirred in to achieve a concentration of 0.15 mM followed by Pepsin (2000 U/mL in the mixture). Gastric digestion was stopped by raising the pH to 7 with NaOH (1 M) after 180 min. The digesta was passed through a sieve pore of 1.4 mm. The filtrate was used for the intestinal digestion. Gastric chyme was mixed with SIF in a 1:1 ratio, bile salts (concentration 10 mM) and CaCl_2_ (0.6 mM). Trypsin (100 U/mL) was added. Digesta (0.5 mL) were collected at 0, 30, 60, 120 and 180 min and frozen immediately in liquid nitrogen. The digesta at time of 0 min were taken out from the reactor immediately after the pepsin was added and mixed with the gastric fluid. All the phases of digestion were carried out at 37 °C under gentle shaking. The digesta samples were mixed with an equal volume of sample buffer with 5% beta-mocaptoethanol before boiling for 5 min, following by analysis with Sodium dodecyl sulfate-polyacrylamide gel electrophoresis (SDS-PAGE) according to Wang et al. [27].

### 2.13. Determination of Free Amino Group

Free amino groups were determined at different time points during gastric and intestinal static digestion according to Mao et al. [8]. One mL of 0.01% 2,4,6-trinitrobenzenesulfonic acid (TNBS) and 2.0 mL (0.2 mol/L, pH 8.2) of phosphate buffer were added to 125 µL of digestive supernatant (digesta were centrifuged and clear supernatants used) followed by mixing and incubation at 50 °C for 30 min while avoiding light. 2 mL of Na_2_SO_3_ (0.1 mol/L) was used to stop the reaction and cooled at room temperature for 15 min. Blank was the replacement of the sample with water under the same conditions. Absorbance was measured at 420 nm. The free amino acid content was determined using the standard curve of L-leucine.

### 2.14. Statistical Analysis

Three independent repeated experiments were conducted at different days using the fresh samples. Analysis of variance (one-way ANOVA) was used to compare means using SPSS Statistics 17 (International Business Machines Corp, Armonk, NY, USA). The effects of treatments and differences between samples were evaluated by Tukey’s test, and a significance level of *p* < 0.05 was selected.

## 3. Results and Discussion

### 3.1. Effect of Ultrasound Pretreatment on Structural Properties of Protein

Intrinsic fluorescence was used to detect if the ultrasound induced changes in the tertiary structure of proteins during treatment. The shift in fluorescence peak to longer wavelengths could indicate that ultrasound treatment made the environment surrounding tryptophan more polar after unfolding. As displayed in Table 1, the ultrasound influenced all ultrasonic samples as emission maxima of intrinsic fluorescence shifted to longer wavelengths and the fluorescence intensity increased at all power levels and frequencies. It suggested that ultrasound induced unwinding in the tertiary protein structure, though all ultrasound pretreatment resulted in little difference with control in emission maxima wavelength and fluorescence intensity (*p* > 0.05). Some similar studies have been reported that small red shift in the maximum fluorescence emission wavelength was able to improve the functionalities of proteins [28,29].

The effect of ultrasonic frequency mode and ultrasonic power on total and free sulfhydryl group (SH) content is listed in Table 1. The total sulfhydryl content was similar with others [30]. With the little unfolding of the protein structure, there was no significant effect on total and free SH content (*p* > 0.05) across all treatments. Our results were consistent with the study of Ren et al. [31] that ultrasound pretreatment for limited time resulted in little change on the total and free SH content of beta-lactoglobulin. It suggested that ultrasonic pretreatment used did not lead to the covalent aggregation of whey protein. As the energy of divergent ultrasound does not focus on small area, treatment intensity on samples using divergent ultrasound is lower than that of gathering ultrasound.

### 3.2. Average Droplet Size of Emulsions Prepared with Ultrasound Modified Whey Protein

As shown in Table 2, ultrasonic frequency mode and ultrasonic power had little significant effect on the average droplet size of the emulsions. It was not surprised because ultrasound was used to pretreat the whey protein before the formation of whey protein in our study. Fine emulsions were prepared by the same procedure of high pressure homogenizer with those whey protein solutions as the emulsifier. Ultrasonic frequency mode and ultrasonic power had showed little effect on structure changes of whey protein. It might lead to little difference in interfacial properties of whey protein. And the similar interfacial properties and same homogenization procedure might result in little impact on the droplet size of the emulsions. Studies have shown the effect of ultrasound to produce significant reduction in size of emulsions [32], which is contrary to the results of this study. The difference was due to the procedure used for preparing the emulsions. In those studies, emulsification was conducted by ultrasound. Changes in the conditions of ultrasound treatment might differ the ultrasonic intensity, and higher input energy induced by higher intensity could result in reduction in size of emulsions.

### 3.3. Zeta-Potential of Emulsions Prepared with Ultrasound Modified Whey Protein

The zeta-potential was studied to observe the interaction between the emulsified droplets in terms of protein aggregation and the role of these droplets to serve as active or inactive fillers in the gel network. The higher values indicated that the droplets had higher repulsion that prevented aggregation. Thus, the emulsion droplets were actively involved as fillers in the formation and strengthening of the gel network [33,34]. Similar to the results of average droplet size, ultrasound treatment demonstrated negligible effect on zeta potential of most of emulsion samples except at the ultrasonic power level of 300 W. The zeta potential of ultrasound treated samples was significantly affected at 300 W for all three frequencies. Higher surface charges recorded for TFU might result from exposure of hidden charges within the native whey as confirmed by intrinsic fluorescence.

### 3.4. Viscosity of Emulsions Prepared with Ultrasound Modified Whey Protein

**Figure 1:**
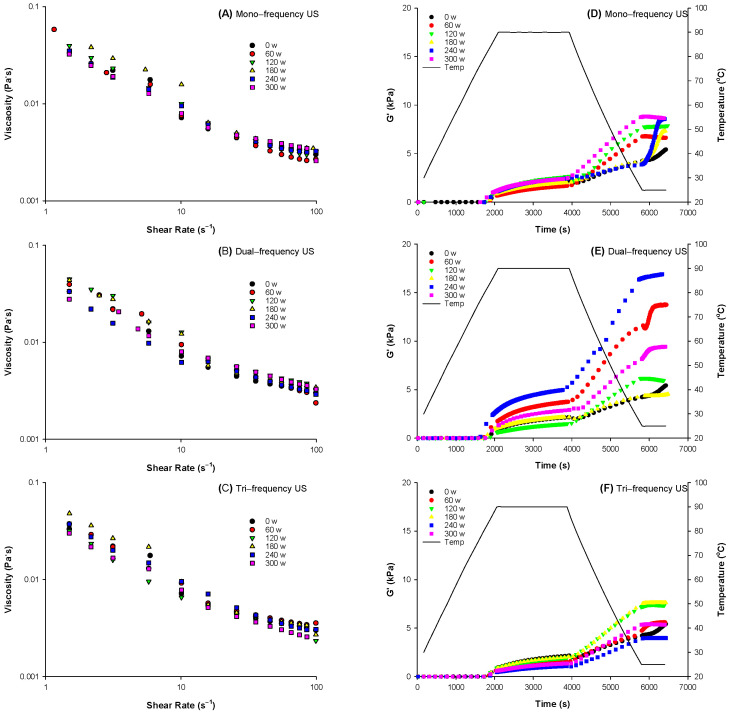
Viscosity (**A**–**C**) of ultrasound pretreated WPI emulsions and storage modulus G′ (**D**–**F**) change in emulsions prepared with whey protein pretreated by ultrasound at different ultrasonic frequency mode and ultrasonic power during heating and cooling cycle.

Figure 1a–c illustrates the shear viscosity of oil-in-water emulsions prepared with ultrasound treated whey protein. All samples exhibited shear thinning behaviour with increasing shear. The viscosity of the emulsions might be affected by the both the emulsified oil droplets and whey protein in bulk phase. The multi-frequency ultrasound treatment caused a shear thinning behaviour in all samples as ultrasound is known to influence the viscosity of protein solutions [35]. All MFU treated samples were thinner than control and became thinner with increasing ultrasonic power and shear (Figure 1A). However, for DFU and TFU, although viscosity was reduced at higher ultrasonic power, some treatments recorded higher values than the control at lower shear stress (TFU-240 W, DFU-60 W and DFU-180 W) (Figure 1B,C). This shear-thinning pattern might be due to the cavitation during the disruption of protein particles into smaller particles at the treated frequency [36].

### 3.5. Effect of Ultrasound Modified Whey Protein on Rheological Properties of WPEG

Emulsions prepared with ultrasound modified whey protein were then used to form the heat-set whey protein emulsion gels (WPEG). Storage modulus (G′) during WPEG formation was studied using a heating and cooling cycle (Figure 1D–F). The ultrasound pretreatment of whey protein improved the G′ value of WPEG except the samples prepared with the whey protein pretreated by DFU at 180 W and TFU at 240 W. There was a higher rate of exponential increment in G′ during cooling across all treatments than heating. The exponential increment in G′ during cooling across all treatments could be attributed to hydrogen and ionic bond formation during cooling.

MFU pretreatment of whey protein produced WPEG with higher G′ than control, and G′ increased with increasing ultrasonic power (Figure 1D). Our results concurs with other studies, where the storage modulus of gels increased after ultrasonic treatment [37,38] and were improved with increasing power intensity at 20 kHz [24]. WPEG with whey protein sonicated at 300 W had the highest G′, which could be due to the maximum cavitation effect at this energy input from mechanical shear and pressure build-up, inducing protein unfolding. The unfolding might promote hydrophobic bonding between non-polar regions that were initially hidden. Pretreatment with DFU at all energy levels improved G′ of the WPEG except 180 W (Figure 1E). DFU at 80% power density (240 W) yielded the highest G′ which were 1.41 times higher than the control. It indicated that WPEG produced by DFU pretreatment was more firm than those by other ultrasonic treatments. WPEG with whey protein pretreated at the ultrasonic power of 180 W gave the highest G′ in the TFU group and G′ declined with increasing ultrasound power (Figure 1F). The decline in G′ of the samples with the treated power level above180 W might be ascribed to the pooled simultaneous cavitation effect of the three operating frequencies. Heating of the TFU samples (beyond 180 W) might resulted in aggregation, which reduced the number of bonds available for gel formation [39].

### 3.6. Effect of Ultrasound Modified Whey Protein on Textural Properties of WPEG

**Figure 2:**
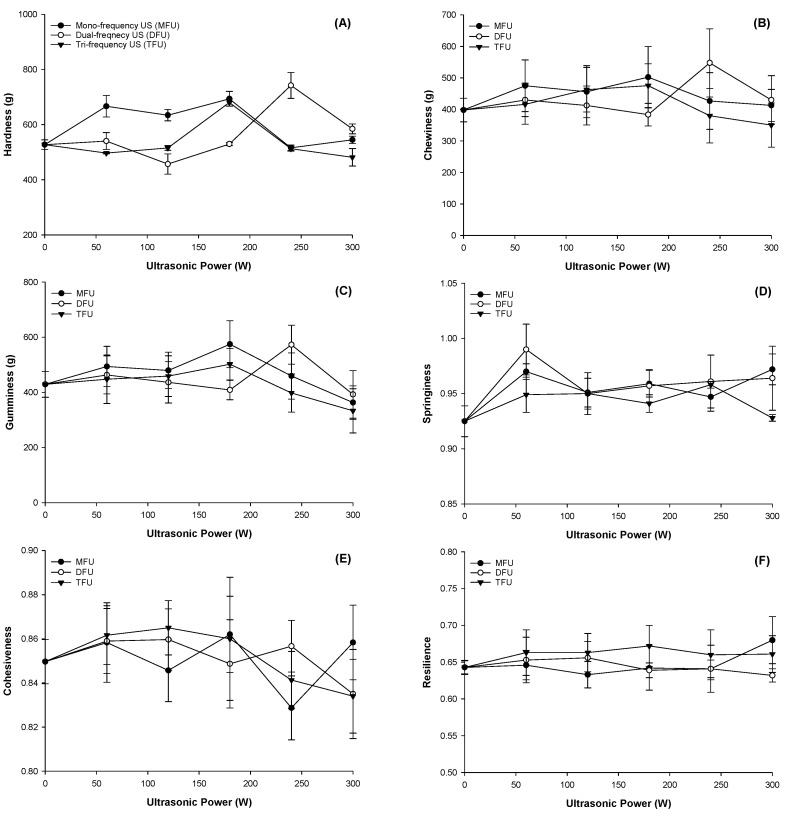
Texture profile analysis of WPEG with whey protein pretreated by ultrasound at different ultrasonic frequency mode and ultrasonic power: (**A**) Hardness; (**B**) Chewiness; (**C**) Gumminess; (**D**) Springiness; (**E**) Cohesiveness; (**F**) Resilience.

The textural characteristics of a product highly influence consumer acceptability and digestion. The TPA was carried out on the WPEG, and findings are displayed in Figure 2. Hardness was significantly affected at all ultrasound power and frequencies (*p* < 0.05) (Figure 2A). Maximum hardness was at the WPEG samples with whey protein pretreated at 180 W for MFU and TFU; and 240 W for DFU. Compared with control, the corresponding increments were of 39.99%, 27.33% and 53.03%, respectively. Gel hardness increased with increasing power in DFU and peaked at 240 W, after which there was a decline. The highest fluorescence intensity at 240 W due to structural unfolding allowed more protein bonds to be available for gel formation. This facilitated protein aggregation during gel formation and thus improved upon hardness and G′ [40]. The ability of DFU to improve on hardness over MFU has been reported in our previous research [15]. The maximum hardness was attained at 180 W for WPEG with TFU pretreatment and then declined with increasing power. This trend concurs with the findings for G′.

Although the ultrasound pretreatment of whey protein produced gels with improved textural properties, DFU pretreatment of whey protein gave the most resilient, springy, cohesive and chewy WPEGs. These observations might be due to different structural changes influenced by the various ultrasound frequencies and pretreatment power on the protein. Chewiness and gumminess followed the pattern exhibited by hardness as the peaks were observed at the WPEG with whey protein pretreated between 180–240 W across all three frequencies (Figure 2B,C). Springiness of WPEG with whey protein pretreated by ultrasound was enhanced at all ultrasonic frequencies and power (*p* > 0.05) with the highest in DFU (*p* < 0.05) followed by MFU (*p* > 0.05) and the least being TFU (*p* > 0.05) (Figure 2D). Results indicated that all gels had good elasticity as they sprang back to over 90% of their original height following compression. WPEG with the whey protein pretreated at all ultrasonic frequencies and power exhibited stronger cohesion as they were all greater than 80% (Figure 2E). WPEG with whey protein treated at 60 W yielded the most resilient products across all frequencies, followed by 300 W (Figure 2F). Gels with the pretreatment of TFU were more resilient than those with other ultrasound treatments. Nonetheless, all samples had resilience beyond 60% (*p* > 0.05).

### 3.7. Effect of Ultrasound Modifyed Whey Protein on WHC of WPEG

**Figure 3:**
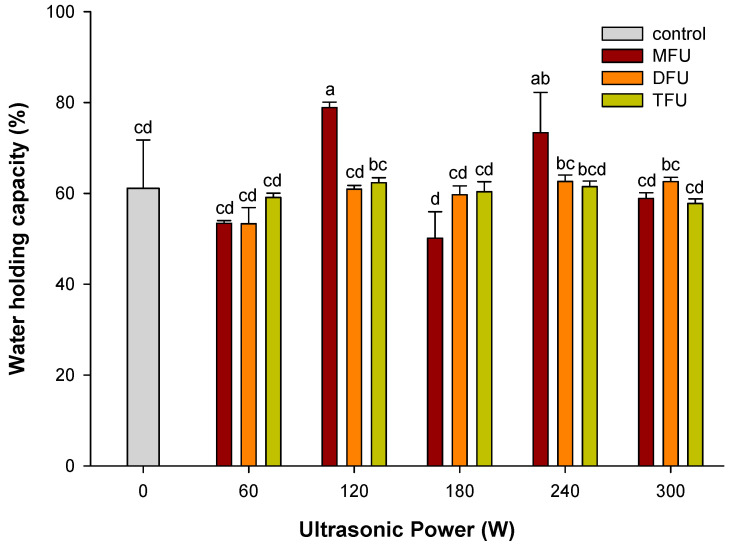
WHC of WPEG with whey protein pretreated by ultrasound at different ultrasonic frequency mode and ultrasonic power. Means with different letters (a–d) differ significantly (*p* < 0.05).

As shown in Figure 3., WPEG with whey protein pretreated by MFU at the power levels of 120 W and 240 W retained more than 70% of water content. WHC of these two samples was enhanced by 29.0% and 20.0% respectively when compared with the control (*p* < 0.05). Structural unfolding of protein during ultrasound treatment coupled with heating of emulsion was known to expose hydrophobic and buried hydrophilic bonds that can affect WHC of gels [38]. The aggregation of protein during gelation with trapped and unbound water might also contribute to WHC of WPEG with whey protein pretreated by DFU and TFU. WHC was reduced to less than 65% in DFU and TFU at all power levels with no significant difference (*p* > 0.05) between the ultrasound and control group. This observation could be attributed to different structural changes induced by the MFU pretreatment at these power levels in the whey protein. The reduction in WHC for gels with whey protein pretreated by DFU and TFU could be due to the poor particulate gels formed during heating [27,41]. After the ultrasound treatment, the shift in wavelengths was around 341–344 nm at all DFU and TFU power levels. This might also have accounted for the minimal differences in WHC between these treatment groups.

### 3.8. Effect of Ultrasound Modifying Whey Protein on Microstructure of WPEG

**Figure 4:**
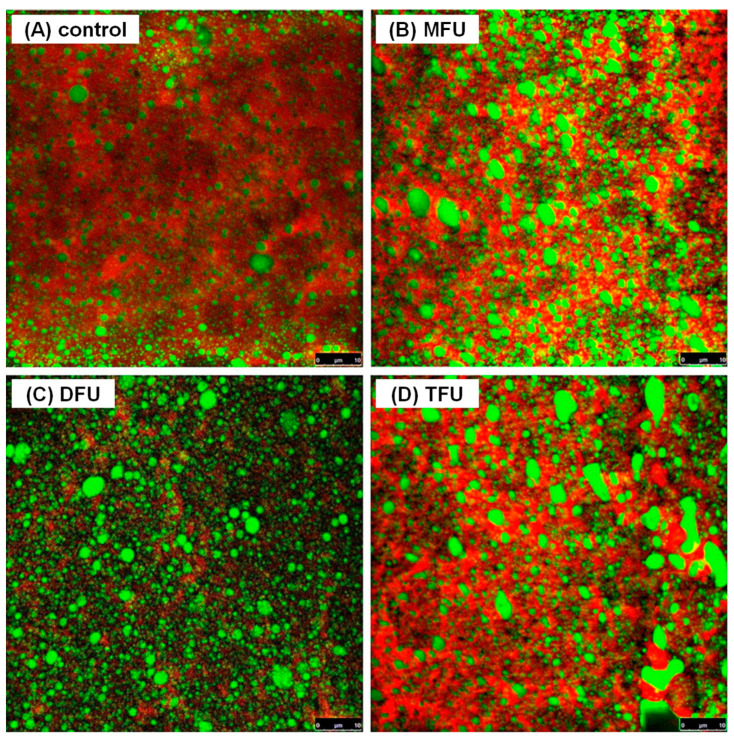
Confocal laser scanning micrographs of WPEG with whey protein pretreated by different ultrasound ((**A**)—control, (**B**)—MFU (300 W), (**C**)—DFU (240 W), (**D**)—TFU (180 W)).

To observe the effect of multi-frequency ultrasound treatments of whey protein on the microstructure of WPEG, the gel morphology was analyzed using CSLM. WPEG with the most improved properties across all treatment groups (MFU-300 W, DFU-240 W and TFU-180 W) were selected and the images are presented in Figure 4. All emulsion gels formed a continuous and denser protein gel network with protein aggregation compared to the control. However, the coalescence of oil droplets was more prominent in the ultrasound treated samples. The distance of the filling emulsified oil droplets in ultrasound pretreated WPEG samples were closer than that in the control. The droplet size of the filling emulsified oil droplets in ultrasound pretreated WPEG samples seemed to be larger. As presented in Figure 4C, WPEG with whey protein pretreated by DFU (240 W) produced a homogenous gel structure with fewer oil coalescence while that by TFU (180 W) yielded less homogenous gel structure with a loose network. WPEG with whey protein pretreated by TFU exhibited a higher degree of protein aggregation during gelation. That partially resulted in the formation of larger protein aggregates. This might explain the softer texture of WPEG produced by TFU pretreatment as shown by hardness analysis. The effect of gel microstructure on gel properties has been reported by other researchers [42,43,44], and our previous results [4,8,15] also confirmed that the homogenous and compact gel network was associated with better gel properties and storage modulus.

### 3.9. Simulated Gastric Digestion and Free Amino Group Release

**Figure 5:**
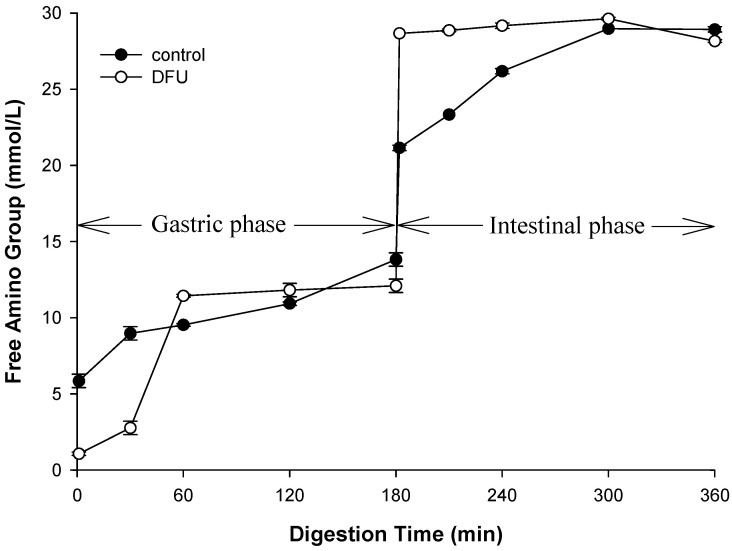
Free amino group released fromcontrol andDFU treated WPEG during simulated gastric digestion.

Simulated digestion was carried out to study the effect of structure of WPEG, induced by the ultrasound on protein hydrolysis. Based on the samples’ structural properties, WPEG with whey protein pretreated by DFU at 240 W (maximum hardness and G′) and control were selected for simulated digestion (Figure S1). The static simulated digestion was monitored at different time points for 180 min in the presence of pepsin at pH 3 with an SDS-PAGE. The results showed that bands of beta-lactoglobulin for both the control and DFU samples persisted through gastric digestion. The control and DFU samples exhibited similar pattern with the literature where beta-lactoglobulin has shown resistance to gastric digestion [45]. The bands of beta-lactoglobulin fade as digestion progressed. The intensity of the bands of beta-lactoglobulin in DFU sample at 30 min was higher than that of control. It suggested the protein digestion of DFU sample was slower than that of control. It was consistent with the results of the release of free amino group (Figure 5). The concentration of the released amino group at 30 min during the gastric phase was slower in the DFU samples than that of control. The WPEG with ultrasound pretreatment produced firmer and harder gels and these properties are known to slow the protein gel hydrolysis rate [46,47,48]. Since the firmer gels with higher storage modulus might demonstrate small porosity. The small size of the porosity might limit the diffusion of pepsin into inner of the gel matrix. The contact of the pepsin with inner whey protein might delay, and the digestion of the whey protein slowed down.

During the gastric digestion period of 30 to 60 min, the release rate of amino group for the DFU samples was higher than that during the initial 30 min and increased quickly. It was not surprised because swelling of the gels during digestion increased the size of gel porosity. The increase in porosity size reduced the diffusion resistance of pepsin. Then pepsin was easy to contact with whey protein, leading to the promotion of protein hydrolysis. On the other hand, ultrasound treatment has been turned out to improve the enzymatic hydrolysis of proteins. DFU treatment on whey protein was able to facilitate protein hydrolysis and lead to increase in the release of free amino group. The similar trend was display in the intestinal phase during the first 60 min. The DFU samples released 33% more free amino groups than the control. Although the free amino groups released in DFU samples were higher than the control in the intestinal phase, the final content of released free amino group showed little difference. Our results suggested DFU pretreatment of whey protein was able to change the digestion kinetics of WPEG. The reason for that might be due to the modification of the microstructure and properties of the WPEG induced by ultrasound pretreatment of whey protein. This changed the diffusion behaviors of pepsin and the affect the contact of pepsin with the whey protein in the gel matrix. Once the enzyme degraded the whey protein, ultrasound pretreatment facilitated the release of free amino group.

## 4. Conclusions

This study highlighted how whey protein pretreated by multi-frequency ultrasound at different power affected WPEG properties. Results confirmed that modifying whey protein at different ultrasonic frequency mode and ultrasonic power was able to affect the textual, rheological properties, WHC and microstructure of WPEG. Ultrasound pretreatment did not show significant difference in tertiary structure and SH content of whey protein. Modifying whey protein with MFU at the power of 120 W and 240 W produced WPEG with higher WHC, whereas modifying whey protein with DFU at the power of 240 W produced WPEG with higher hardness and storage modulus. WPEG prepared with whey protein modified by DFU at the power of 240 W demonstrated more homogenous and compact gel network, which resulted in the increase in gel firmness and higher G′ with smaller porosity. The network might hinder the diffusion of pepsin into gel matrix to decrease protein hydrolysis rate. Therefore, release of free amino group from WPEG with whey protein pretreated by DFU was slower than that of the control. Our findings provide new insights for designing protein emulsion gels with specific properties and digestion rate with multi-frequency ultrasound.

## Figures and Tables

**Table 1:** Effect of US frequency and power on sulphydryl content and intrinsic fluorescence (n = 3).

Power Level (W)	Frequency (kHz)	SH (µmol/g Protein)		Intrinsic Fluorescence	
		Free	Total	λ_max_ (nm)	Intensity
0	0	5.74 ± 1.08 ^a^	20.34 ± 1.85 ^A^	338.9 ± 3.5 ^a^	244.6 ± 2.2 ^A^
60	20	7.40 ± 1.78 ^a^	21.96 ± 2.73 ^A^	343.1 ± 1.4 ^a^	244.6 ± 3.7 ^A^
	20/35	7.46 ± 2.23 ^a^	19.76 ± 1.84 ^A^	342.2 ± 1.4 ^a^	248.8 ± 4.2 ^A^
	20/35/50	7.68 ± 1.17 ^a^	17.64 ± 3.46 ^A^	340.4 ± 0.3 ^a^	249.9 ± 4.2 ^A^
120	20	6.67 ± 2.55 ^a^	20.29 ± 2.08 ^A^	341.4 ± 3.2 ^a^	251.4 ± 1.9 ^A^
	20/35	7.36 ± 2.15 ^a^	20.31 ± 2.97 ^A^	340.8 ± 3.0 ^a^	239.5 ± 2.0 ^A^
	20/35/50	8.34 ± 0.98 ^a^	21.02 ± 2.57 ^A^	344.3 ± 2.1 ^a^	256.4 ± 4.3 ^A^
180	20	6.39 ± 2.16 ^a^	21.75 ± 2.99 ^A^	341.3 ± 1.4 ^a^	251.8 ± 3.9 ^A^
	20/35	6.80 ± 2.04 ^a^	20.66 ± 3.25 ^A^	342.2 ± 2.8 ^a^	246.2 ± 3.2 ^A^
	20/35/50	7.18 ± 2.03 ^a^	20.96 ± 2.02 ^A^	341.1 ± 3.1 ^a^	266.8 ± 2.9 ^A^
240	20	7.23 ± 1.85 ^a^	22.21 ± 2.75 ^A^	342.1 ± 0.3 ^a^	248.7 ± 5.4 ^A^
	20/35	6.26 ± 1.42 ^a^	18.46 ± 2.01 ^A^	340.4± 2.0 ^a^	247.1 ± 3.8 ^A^
	20/35/50	7.28 ± 0.34 ^a^	17.60 ± 2.76 ^A^	341.5 ± 2.0 ^a^	251.1 ± 2.1 ^A^
300	20	7.70 ± 1.70 ^a^	20.29 ± 2.37 ^A^	341.9 ± 1.5 ^a^	248.8 ± 5.3 ^A^
	20/35	7.54 ± 2.25 ^a^	20.52 ± 1.72 ^A^	341.6 ± 2.3 ^a^	246.2 ± 0.7 ^A^
	20/35/50	8.17 ± 1.39 ^a^	22.83 ± 3.55 ^A^	341.0 ± 1.8 ^a^	256.8 ± 4.0 ^A^

Means in the same column that share thesame letters (a or A) are not significantly different.

**Table 2:** Droplet size and zeta potential of the emulsions prepared with whey protein modified by ultrasound at different frequency and power (n = 3).

Power Level (W)	Frequency (kHz)	Droplet Size (nm)	Zeta Potential (mV)
0	0	382.2 ± 68.9 ^a^	26.0 ± 1.6 ^abc^
60	20	472.7 ± 80.2 ^a^	27.0 ± 2.0 ^abc^
	20/35	426.3 ± 72.6 ^a^	25.7 ± 1.6 ^abc^
	20/35/50	436.1 ± 169.1 ^a^	27.6 ± 1.0 ^abc^
120	20	372.7 ± 50.4 ^a^	27.9 ± 1.2 ^abc^
	20/35	480.4 ± 87.8 ^a^	26.9 ± 1.4 ^abc^
	20/35/50	397.3 ± 3.2 ^a^	27.1 ± 2.0 ^abc^
180	20	383.4 ± 59.6 ^a^	25.7 ± 2.8 ^abc^
	20/35	470.5 ± 26.0 ^a^	25.8 ± 0.8 ^abc^
	20/35/50	390.0 ± 36.7 ^a^	28.9 ± 0.6 ^bc^
240	20	404.1 ± 38.7 ^a^	26.0 ± 2.2 ^abc^
	20/35	532.6 ± 136.6 ^a^	25.9 ± 1.2 ^abc^
	20/35/50	419.6 ± 81.1 ^a^	27.8 ± 1.0 ^abc^
300	20	415.5 ± 84.2 ^a^	24.8± 1.6 ^ab^
	20/35	402.2 ± 48.7 ^a^	23.8 ± 2.0 ^a^
	20/35/50	332.8 ± 22.5 ^a^	29.0 ± 2.2 ^c^

Means with difference letters (a–c) are significantly different.

## Data Availability

Not applicable.

## Supplementary Materials

The following supporting information can be downloaded at: https://www.mdpi.com/article/10.3390/polym14102054/s1, Figure S1: SDS-PAGE of (A) control and (B) DFU treated WPEG under gastric digestion at different time digestion points.
