# Supplementary material for: Gelling Characteristics of Emulsions Prepared with Modified Whey Protein by Multiple-Frequency Divergent Ultrasound at Different Ultrasonic Power and Frequency Mode
Source: Polymers (Basel). 2022 May 18;14(10):2054. doi: 10.3390/polym14102054 (PMC9144504; doi:10.3390/polym14102054)
Supplement: Supplementary file 1 [file polymers-14-02054-s001.zip › polymers-1666778-supplementary.pdf]

Supplementary Materials

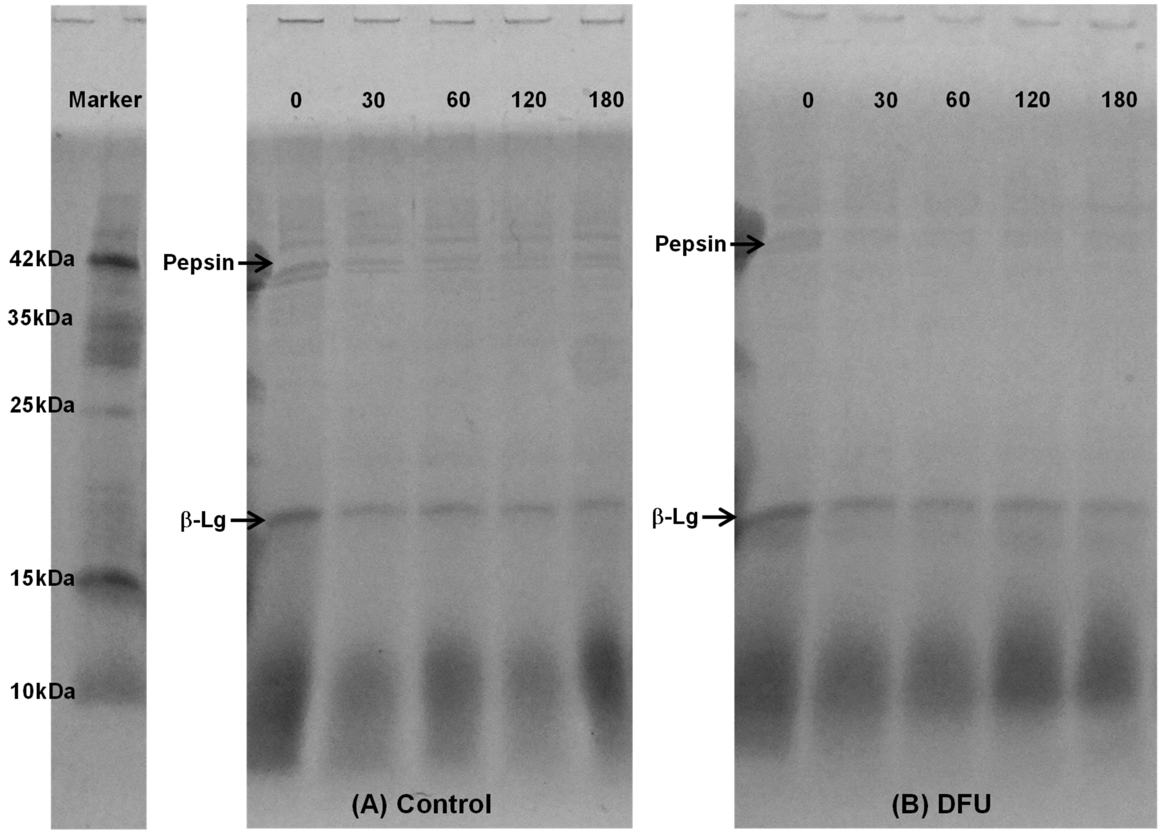

Figure S1. SDS-PAGE of (**A**) control and (**B**) DFU treated WPEG under gastric digestion at different time digestion points.
